# Relationship between binge drinking experience and suicide attempts in Korean adolescents: based on the 2013 Korean Youth Risk Behavior Web-based Survey

**DOI:** 10.4178/epih.e2018046

**Published:** 2018-09-26

**Authors:** Kyeong Hyang Byeon, Sun Ha Jee, Jae Woong Sull, Bo Young Choi, Heejin Kimm

**Affiliations:** 1 Department of Public Health, Graduate School, Hanyang University, Seoul, Korea; 2 Department of Epidemiology and Health Promotion, Graduate School of Public Health, Yonsei University, Seoul, Korea; 3 Department of Biomedical Laboratory Science, Eulji University College of Health Sciences, Seongnam, Korea; 4 Departments of Public Health and Medical Administration, Dongyang University, Yeongju, Korea

**Keywords:** Korean adolescents, Binge drinking experience, Suicide attempted, Korea

## Abstract

**OBJECTIVES:**

Suicide and drinking problems in adolescents are increasing every year, and it is known that suicide is related to drinking. This study aims to identify the relationship between binge drinking experience (BDE) and suicide attempts in Korean adolescents.

**METHODS:**

The Ninth Korean Youth Risk Behavior Web-based Survey (KYRBS), conducted in 2013, was used for analysis. Multiple logistic regression analysis was used to identify the relationship between BDE and suicide attempts, and the relationship between BDE and suicide attempts in middle and high school students was stratified by age.

**RESULTS:**

BDE and suicide attempts were highly related. The odds ratio (OR) of attempted suicide in BDE was 1.63 times (95% confidence interval [CI], 1.28 to 2.09) higher then non-drinking in males. And the OR of attempted suicide in females was 1.21 times (95% CI, 1.07 to 1.37) higher then non-drinking in non-BDE, 1.79 times (95% CI, 1.47 to 2.19) higher in BDE. BDE was associated with suicide attempts in males aged 12 or 13 years (OR, 3.97; 95% CI, 1.57 to 10.03) and in females aged 15 years (OR, 2.66; 95% CI, 1.79 to 3.96).

**CONCLUSIONS:**

BDE is an important factor related to suicide attempts in adolescents. In order to reduce suicide attempts, it is necessary to educate the youth about the regulation of BDE and drinking prevention.

## INTRODUCTION

The leading cause of death among Korean adolescents in 2011 was intentional self-harm (suicide), and the number of adolescent suicides per 100,000 population continued to increase from 7.7 in 2001 to 13.0 in 2011. In addition, 11.2% of Korean adolescents in 2012 reported having thoughts about wanting to commit suicide at least once in the past year [1]. The rate of adolescent suicide attempts steadily increased from 2006 to 2013, 4.1% in 2013 and showed a declining trend to 2.9% from 2014 onwards [2].

Suicide attempts are in the middle of a continuum from suicidal ideation to suicide attempts and suicide [3]. People who enact suicidal behavior usually have suicide ideation, those who have suicidal ideation make suicide attempts, and a successful suicide attempt results in a completed suicide [4]. In order to prevent suicide attempts in adolescents, it is important to look at the current status of suicide attempts among adolescents, and it is also necessary to identify the factors affecting suicide attempts among adolescents.

Although it has received less attention in Korean, foreign studies have highlighted binge drinking as a factor related to suicide attempts in adolescents [5,6]. Most adolescents have drinking experiences, and the number of adolescents exhibiting risky drinking behaviors, such as binge drinking or habitual drinking beyond the stage of drinking out of curiosity, is increasing among adolescents who drink alcohol [7]. From 2006 to 2012, the risky drinking rate in Korea was reported to be high, over 10.3% in males and over 8.0% in females [2]. Binge drinking in adolescents is very important as a starting point for problem behaviors, such as alcohol consumption or drinking behavior [8], and can easily lead to adverse outcomes such as addiction, serious health problems, injury, traffic accidents, and suicide [9,10]. In addition, binge drinking in adolescents has been reported to be associated with depression among psychiatric disorders, thereby affecting suicide attempts [5,11-13]. Adolescents with BDE were found to have mental health problems, including mood disorders and anxiety disorders. Regarding the relationship between alcohol consumption and suicidal behavior, drunkenness was found to increase the risk of suicide by about 90 times [14], and binge alcohol consumption was found to increase the risk of suicide by about 5 times compared with social drinking [15].

Recent studies regarding the relationship between drinking and suicide attempts as a factor for adolescent suicide have shown early alcohol use initiation as a risk factor for suicide attempts [16-18]. Compared to non-drinking or alcohol use initiation after the age of 13 years, there was a statistically significant association between early alcohol use initiation before the age of 13 years and suicide attempts among adolescents [18,19]. Early alcohol use initiation among adolescents was found to be a risk factor affecting suicide attempts [20].

There have been foreign studies regarding binge drinking, and binge drinking among young adolescents was found to be a risk factor for suicide attempts [6]. However, domestic studies on suicide attempts related to binge drinking among drinking behavior factors in adolescents are scarce. This study aims to investigate the relationship between BDE, known as risky drinking, and suicide attempts among Korean adolescents, stratified by age.

This study aims to investigate the relationship between BDE and suicide attempts in Korean adolescents, to understand the factors for suicide attempts, and to use this as baseline data regarding adolescent binge drinking and for mental health promotion.

The specific objectives are as follows: First, to investigate the relationship between non-drinking, non-BDE, and BDE and suicide attempts in adolescents. Second, to investigate the relationship between BDE and suicide attempts in adolescents, stratified by age.

## MATERIALS AND METHODS

### Research data and subjects

This study was conducted using raw data from the 2013 Korean Youth Risk Behavior Web-based Survey (KYRBS) which was conducted jointly by the Ministry of Education, Science and Technology, Ministry of Health and Welfare Affairs, and Korea Centers for Disease Control and Prevention. The KYRBS has been conducted annually since 2005 and is considered to be a nationally representative school youth sample. The subjects were middle and high school students from first-year middle school students to third-year high school students across the country, classified by 16 cities and provinces into metropolitan cities, medium and small cities, and *gun* (rural area). For the sampling, the primary sampling unit was school and the secondary sampling unit was class. All the students in the selected sample of classes were surveyed and students with long-term absences, exceptional children, or students with letters decoding disability were excluded from the sample. The survey questionnaire consists of 102 items in 15 domains including tobacco use, alcohol use, obesity/weight control, physical activity, dietary behaviors, prevention of injury, sexual behaviors, mental health, Internet addiction, oral health, atopic dermatitis, asthma, personal hygiene, substance use, and health equity. For the survey method, one computer per student was assigned in each sampled classroom in a computer laboratory where Internet was available. The students were randomly seated to anonymously complete the self-administered online survey. The 9th survey (2013) was conducted with 75,149 students from 800 schools, including 400 middle schools and 400 high schools. In total, 72,435 students from 799 schools participated in the survey (96.4%) [21]. The subjects of the 8th KYRBS (2012) were a total of 75,149 students from 800 schools, including 400 middle schools and 400 high schools. In total, 72,435 students from 799 schools participated in the survey with a response rate of 96.4% [21].

Among the surveyed middle and high school students, 40,622 students without drinking experience reported not having a lifetime drinking experience; 26,105 students with non-BDE reported having a lifetime drinking experience, but not having a drinking experience during the past one month. Other students reported that their average drink consumption was 1-2 cups of soju (Korean distilled liquor) for females and either 1-2 or 3-4 cups of soju for males if they consumed alcohol during the past month. Among the 5,708 students with BDE, reporting that their average drink consumption was more than 3-4 cups of soju for females and more than 5-6 cups of soju for males during the past month, were selected for final analysis in this study.

### Selection and definition of variables

The main variables in this study, aimed to investigate the relationship between BDE and suicide attempts, are as follows.

#### Suicide attempts

As a dependent variable in this study, a suicide attempt was defined as a person who reported having attempted suicide during the past year. Subjects were then classified into having attempted suicide during the past year or not.

#### Non-drinking, non-binge drinking, and binge drinking experiences

In the present study, those without drinking experience were defined as those who reported not having a drinking experience in their entire lifetime. In addition, those with non-BDE are those who reported not having drank more than one cup of an alcoholic beverage during the last month and who reported that their average consumption per drinking occasion in the last month was 1-2 cups of soju for females and was either 1-2 cups of soju or 3-4 cups of soju for males. Those with BDE were defined as females and males who drank at least 3-4 cups of alcoholic beverages and 5 cups of alcoholic beverages per drinking occasion respectively, regardless of alcohol beverage type, and were thus defined as those drinking more than a single cup of alcoholic beverage per drinking occasion [9,22].

In the present study, BDE is related to a question about alcohol consumption quantity, and was defined as those who reported that their average drink consumption per drinking occasion during the past month was more than 3-4 cups of soju (2 bottles of beer, 3 glasses of liquor) for females and was more than 5 cups of soju (3 bottles of beer, 5 glasses of liquor) for males.

#### Demographic and socioeconomic factors

Age, residential area, grade level, and academic performance were used as demographic factors. The residential area was divided into large cities, medium and small cities, and *gun* areas. Academic performance was classified into high, middle, and low grades in the last year. Socioeconomic factors were divided into high, middle, and low using Family Affluence Scale scores.

#### Health behavior and mental health factors

Smoking and substance experience were used as health behavior factors. Smoking and substance experience were classified by responding with a “yes” or “no” to each question about smoking and substance experience. Stress, depression, suicidal ideation, and suicide plans were used as mental health factors. Stress was classified as feeling a lot of stress, feeling a little bit of stress, and feeling no stress. Depression experience was classified by responding with a “yes” or “no” as to whether the participants had felt sad or desperate enough to interrupt their daily life for two weeks during the past year. Suicidal ideation and suicide plans were classified by responding with “yes” and “no” to a question asking whether each participant had considered seriously suicide and had set up a suicidal plan during the last year.

### Statistical analysis

The data from the KYRBS is complex sample survey data and it was analyzed by employing weight, cluster, and stratification in the process of statistical analysis.

In this study, a chi-square test was performed to examine the general characteristics of those with non-drinking, those with non-BDE and those with BDE, BDE, and suicide attempts, and a significant difference between binge drinking and suicide attempts among those with BDE stratified by age. In addition, a logistic regression analysis was performed to investigate the relationship between non-drinking, non-heaving drinking experience, BDE and the relationship between BDE and suicidal attempts in adolescents stratified by age, and association between binge drinking and suicide attempts, and to calculate the odds ratios (ORs) and 95% confidence intervals (CIs).

All the analyses were performed using SAS version 9.2 (SAS Institute Inc., Cary, NC, USA). The statistical significance level was set at p-value<0.05.

## RESULTS

### General characteristics of those with binge drinking experience

In terms of grade level, a demographic factor in males, the proportion of non-drinkers was high in all the years of middle school students and the first-year high school students, whereas the proportion of those with non-BDE was high in the second- and third-year high school students, and there was a statistically significant difference (p<0.001) (Table 1). In terms of socioeconomic factors, the proportion of non-drinkers was high in all those with a high, middle, or low score on the Family Affluence Scale, and there was a statistically significant difference (p<0.001). Concerning the health behavior factors, 56.4% of those with smoking experience had non-BDE and 23.2% of those with smoking experience had BDE with a high proportion, and there was a statistically significant difference (p<0.001). In addition, 36.7% of those with substance experience had BDE, whereas 34.5% of them had non-BDE with a high proportion, and there was a statistically significant difference (p<0.001). In terms of mental health factors, 45.0% of those with depression experience had non-BDE with a high proportion. In addition, those with suicidal ideation and suicide plans had more non-BDE, and the proportion of those with non-BDE among those with suicide attempts was high with 42.0%, showing a statistically significant difference (p<0.001).

Meanwhile, regarding grade level, a demographic factor in females, the proportion of non-drinkers was high in all the middle school students and the first- and second-year high school students. Additionally, the proportion of those with non-BDE was high with 45.1% in the third-year high school students, showing a statistically significant difference (p<0.001). The proportion of non-drinkers was high in all those with a high, middle, or low score on the Family Affluence Scale and there was statistically significant difference (p<0.001).

Concerning the health behavior factors, 54.7% of those with smoking experience had non-BDE, whereas 30.5% of them had BDE with a high proportion, and there was a statistically significant difference (p<0.001). In addition, 45.1 and 29.5% of those with substance experience had BDE and non-BDE, respectively, with a high proportion, and there was a statistically significant difference (p<0.001). Concerning the mental health factors, the proportion of non-drinkers was high in both those with depression and those without depression experience, and there was a statistically significant difference (p<0.001). In addition, 47.8% of those with suicidal ideation were non-drinkers, whereas 40.8% of them had non-BDE, and 44.6% of those with suicidal plan were non-drinkers, whereas 40.8% of them had non-BDE, and there was a statistically significant difference (p<0.001). Further, 42.9% of those with suicidal attempts were non-drinkers, whereas 40.7% of those had non-BDE with a high proportion, and there was a statistically significant difference (p<0.001).

### Binge drinking experience and suicide attempts

In males, 2.0% of non-drinkers, 3.0% of those with non-BDE, and 6.9% of those with BDE attempted suicide, showing a significance difference between the groups (p<0.001) (Table 2).

In females, 3.9% of non-drinkers, 7.0% of those with non-BDE, and 14.2% of those with BDE attempted suicide, showing a significant difference between the groups (p<0.001).

Looking at BDE and suicide attempts in males with BDE according to age, 17.1% of those with BDE attempted suicide at the age of 12 or 13 years, 15.4% at the age of 14 years, and 7.8% at the age of 15 years, 4.6% at the age of 16 years, 4.6% at the age of 17 years, and 5.7% at the age of 18 years, showing a significant (p<0.001) (Table 3).

In females with BDE, 30.6% of those with BDE attempted suicide at the age of 12 or 13 years, 26.6% at the age of 14 years, 21.5% at the age of 15 years, 10.4% at the age of 16 years, 9.0% at the age of 17 years, and 7.9% at the age of 18 years, showing a significant difference (p<0.001) (Table 3).

### Relationship between binge drinking experience and suicide attempts

After adjusting from age, health behavior factors and mental health factors in males, the adjusted OR of suicide attempts in those with non-BDE compared to non-drinkers was 1.04 times (95% CI, 0.88 to 1.23), showing no significant difference. The adjusted OR of suicide attempts in those with BDE was 1.63 times (95% CI, 1.28 to 2.09), showing a significant difference (Table 4).

Meanwhile, after adjusting for age, health behavior factors and mental health factors in females, the odd ratio of suicide attempts in those with non-BDE was 1.21 times (95% CI, 1.07 to 1.37) compared to the non-drinkers, and the OR of suicide attempts in those with BDE was 1.79 times (95% CI, 1.47 to 2.19), showing significant differences.

### Relationship between binge drinking experience and suicide attempts by age

After adjusting for health behavior and mental health factors in males, the OR of suicide attempts in those with BDE at age of 12 or 13 years compared to non-BDE was 3.97 times (95% CI, 1.57 to 10.03), the OR of suicide attempts in those with BDE at age of 14 years was 3.21 times (95% CI, 1.80 to 5.71), and the OR of suicide attempts in those with BDE at age of 18 years was 2.32 times (95% CI, 1.07 to 5.03), and all showed significant differences (Table 5). In males, the OR of suicide attempts in those with BDE at the age of 12 or 13 years compared to non-BDE was found to be high.

Meanwhile, after adjusting for health behavior and mental health factors in females, the OR of suicide attempts in those with BDE at the age of 15 years compared to non-BDE was 2.66 times (95% CI, 1.79 to 3.96), and the OR of suicide attempts in those with BDE at the age of 16 years compared to non-BDE was 1.61 times (95% CI, 1.09 to 2.36), and all showed significant differences. In female, the OR of suicide attempts in those with BDE at the age of 15 years compared to non-BDE was found to be high.

## DISCUSSION

Adolescent suicide and suicidal behavior are a major concern for many countries. Suicide behavior is classified as suicidal ideation, suicide attempts, and suicide [23]. Adolescent suicide is a serious problem. In 2012, 11.2% of adolescents reported having suicidal ideation, which increased by 2.4% from 2010. According to the data from the 2013 KYRBS, 4.1% of adolescents reported having attempted suicide [1]. According to the current status of alcohol use among Korean adolescents, the current prevalence of adolescent alcohol use is 21.1%; 47.2% of adolescents with alcohol use is engaged in risky drinking, and 1 in 5 adolescents has drunken enough to be intoxicated [24]. Although the prevalence of adolescent drinking is decreasing, the proportion of adolescents who are engaged in risky drinking and problem drinking is considerable, and the drinking initiation age became slightly higher [24]. As such, suicidal behavior and drinking-related problems in adolescents are increasing every year. In foreign countries, binge drinking or risky drinking has been pointed out as a risk factor related to adolescent suicide, and binge drinking has been reported as a factor affecting suicidal behavior.

In this study, we analyzed the relationship between BDE and suicide attempts in adolescents using the data from the 2013 KYRBS. The results of this study found that the OR of suicide attempt was high in male and female adolescents with BDE, which is consistent with the results of a previous study indicating that BDE in adolescents was an important factor related to suicide attempt [23]. However, there is a difference in the definition of BDE between this study in which BDE was defined by the drinking quantity and the previous study in which it was defined by the number of drinking days. In addition, this study also found that there was a relationship between suicide attempts and BDE even after controlling smoking experience and drug experience as risk factors for suicide attempt. This suggests that adolescents who are engaged in binge drinking are likely to be led to suicidal behaviors more easily owing to decreased cognitive and problem-solving abilities and increased impulsive behaviors. In addition, their deteriorated ability to control emotions due to binge drinking is likely to lead to negative emotions, and a lack of rational judgment is also an important factor in making the decision to attempt suicide [25]. Therefore, binge drinking in adolescent was found to be a factor affecting the behavior to attempt suicide, and it is thus necessary to strictly control adolescent alcohol drinking at the national level so that it is not easy for adolescents to drink alcohol, and appropriate sanctions and management are needed in the community when drinking adolescents are seen.

As for the relationship between BDE and suicide attempts in middle and high school students stratified by age, the relationship between BDE and suicide attempts was found to be high in male adolescents aged 12 and 13 years old, and female adolescents aged 15 years old with BDE, compared to non-BDE, which is consistent with the results of a previous study reporting that BDE in young adolescents was a strong risk factor for adolescent suicide attempts compared to non-BDE [6]. In the previous study, adolescents were not stratified by sex, but adolescents as a whole were studied. As aforementioned, the OR of suicide attempts was high in middle school students with BDE. Early adolescence is a time when young adolescents have a confusion of value, their self-identity is less established yet, and they are unstable because they are cognitively immature. Early binge drinking initiation in such adolescence acts as an accelerator in suicide attempts, and has a direct adverse effect on health outcomes such as alcohol dependence, drug use, sexual experience, and violence. A previous study reported that early alcohol drinking initiation is one of major factors in the possible development of alcohol-related disorders after adolescence and serves as a risk factor for adulthood alcohol dependence [16]. Binge drinking in early adolescence acts as an accelerator in the stage of experiencing mental and physical changes, thereby leading to suicide, which is an impulsive behavior. Therefore, it is necessary to educate about prevention of alcohol drinking and binge drinking in early adolescence.

A previous study showed that adolescents made more suicide attempts when they experienced depression and/or suicidal ideation, and these were observed to be risk factors affecting suicide attempts [26]. It is thought that experience of depression and suicidal ideation along with heaving drinking experience in adolescence may lead to impulsive suicidal behavior. Therefore, for students with depression, early detection of depression is required through depression screening. Students with suicidal ideations have to be screened in advance through counseling, and education and programs about mental health factors related to suicide attempts and alcohol abstinence should be introduced and provided to them.

Previous studies regarding adolescent drinking and suicide attempts included a study regarding drinking experience as one of factors associated with suicide attempts, a study regarding the effects of drinking behavior on suicidal ideation, and a study regarding the relationship between drinking and suicidal behavior [23,26,27]. However, this study investigated the relationship between BDE and suicide attempts, and found that the OR of suicide attempts was high in young adolescents with BDE.

This study has limitations. Because this study is a cross-sectional study using secondary data, this study cannot identify the causal relationship between BDE and suicide attempts. Another limitation is that because the relationship between binge drinking and suicide attempt was investigated using given variables, various binge drinking behavior such as the initiation of binge drinking and days of binge drinking were not measured in this study. In addition, since the data from the KYRBS is based on a self-administered survey, there is a possibility for the participants to respond in an underestimated or overestimated way than it actually was.

Nevertheless, this study analyzed the relationship between BDE and suicide attempts using the data from a nationally representative school-aged adolescent sample in Korea. It found that adolescent binge drinking increased the OR of adolescent suicide attempts and that young adolescents with BDE increased the OR of suicide attempts. In order to prevent suicide attempts in younger adolescents, it is necessary to regulate binge drinking and to recognize the danger of drinking behavior among adolescents, and health education at schools about drinking abstinence should be periodically conducted.

## Figures and Tables

**Table 1.** General demographic, socioeconomic, and health-related factors in Korean adolescents

Variables	Male			p-value	Female			p-value
	Non-drinking	Non-BDE	BDE		Non-drinking	Non-BDE	BDE	
Demographic factor								
Residence								
Rural area	1,395 (48.0)	1,200 (40.1)	381 (11.9)	<0.01	1,489 (56.8)	956 (35.6)	211 (7.5)	0.09
Metropolis	10,196 (53.6)	7,095 (38.3)	1,478 (8.2)		11,389 (62.3)	5,849 (31.7)	1,144 (6.0)	
Medium cities	7,503 (50.5)	6,025 (40.5)	1,382 (9.0)		8,650 (60.9)	4,980 (32.3)	1,112 (6.7)	
Grade level								
Middle school 1	4,715 (74.3)	1,665 (25.2)	31 (0.5)	<0.001	4,545 (79.0)	1,197 (20.2)	46 (0.8)	<0.001
Middle school 2	4,196 (67.0)	1,946 (31.1)	119 (1.9)		4,276 (73.9)	1,423 (23.6)	153 (2.6)	
Middle school 3	3,598 (59.0)	2,363 (36.5)	288 (4.5)		4,025 (69.1)	1,690 (27.1)	254 (3.8)	
High school 1	2,827 (47.0)	2,658 (43.7)	613 (9.3)		3,448 (59.3)	2,092 (34.7)	390 (6.0)	
High school 2	1,958 (35.8)	2,708 (48.6)	929 (15.5)		2,883 (47.3)	2,618 (40.8)	769 (11.9)	
High school 3	1,800 (31.2)	2,980 (49.4)	1,261 (19.4)		2,351 (41.9)	2,765 (45.1)	855 (12.9)	
Academic achievement								
High	7,477 (58.1)	4,587 (35.6)	820 (6.3)	<0.001	7,929 (67.0)	3,579 (28.4)	603 (4.6)	<0.001
Middle	5,181 (51.9)	3,949 (40.5)	767 (7.6)		6,417 (64.3)	3,266 (30.6)	568 (5.1)	
Low	6,436 (46.2)	5,784 (41.9)	1,654 (11.8)		7,182 (54.4)	4,940 (36.6)	1,296 (9.0)	
Socioeconomic factors								
Economic status of family								
High (6-9)	7,055 (53.1)	5,127 (38.8)	1,098 (8.1)	<0.001	8,051 (63.9)	3,989 (30.1)	807 (5.9)	<0.001
Middle (3-5)	9,648 (50.6)	7,600 (40.6)	1,669 (8.8)		11,175 (60.5)	6,378 (33.2)	1,297 (6.3)	
Low (0-2)	2,391 (54.5)	1,593 (35.3)	474 (10.2)		2,302 (57.9)	1,418 (33.7)	363 (8.4)	
Health behavior factors								
Smoking experience								
No	16,843 (65.3)	8,152 (32.1)	658 (2.6)	<0.001	20,884 (68.0)	9,297 (28.9)	1,006 (3.0)	<0.001
Yes	2,251 (20.4)	6,168 (56.4)	2,583 (23.2)		644 (14.8)	2,488 (54.7)	1,461 (30.5)	
Substance experience								
No	18,957 (52.3)	14,157 (39.4)	3,083 (8.3)	<0.001	21,457 (61.8)	11,701 (32.1)	2,344 (6.1)	<0.001
Yes	137 (28.9)	163 (34.5)	158 (36.7)		71 (25.4)	84 (29.5)	123 (45.1)	
Mental health factors								
Stress								
Feeling a lot of stress	5,684 (45.6)	5,370 (43.2)	1,447 (11.2)	<0.001	9,438 (54.8)	6,647 (36.8)	1,606 (8.4)	<0.001
Feeling a little bit of stress	8,457 (52.8)	6,274 (39.5)	1,250 (7.7)		9,112 (67.1)	4,070 (28.3)	678 (4.6)	
Do not feel stress	4,953 (60.3)	2,676 (33.0)	544 (6.7)		2,978 (70.8)	1,068 (25.0)	183 (4.2)	
Depression experience								
No	15,354 (55.7)	10,192 (37.4)	1,916 (6.9)	<0.001	14,894 (67.4)	6,609 (28.3)	1,040 (4.2)	<0.001
Yes	3,740 (40.9)	4,128 (45.0)	1,325 (14.1)		6,634 (51.4)	5,176 (38.5)	1,427 (10.1)	
Suicidal ideation								
No	17,198 (53.8)	12,143 (38.3)	2,544 (7.9)	<0.001	18,127 (65.0)	8,785 (29.9)	1,568 (5.1)	<0.001
Yes	1,896 (40.1)	2,177 (45.9)	697 (14.0)		3,401 (47.8)	3,000 (40.8)	899 (11.5)	
Suicide plan								
No	18,393 (52.6)	13,586 (39.2)	2,921 (8.2)	<0.001	20,476 (62.7)	10,796 (31.5)	2,095 (5.8)	<0.001
Yes	701 (40.0)	734 (42.2)	320 (17.8)		1,052 (44.6)	989 (40.8)	372 (14.7)	
Suicide attempts								
No	18,698 (52.4)	13,877 (39.2)	3,015 (8.3)	<0.001	20,711 (62.6)	10,985 (31.6)	2,128 (2.8)	<0.001
Yes	396 (36.8)	443 (42.0)	226 (21.2)		817 (42.9)	800 (40.7)	339 (16.4)	

Values are presented as number (%). BDE, binge drinking experience.

**Table 2.** Binge drinking experience (BDE) and suicide attempts

Variable	Total (n)	Male		p-value	Total (n)	Female		p-value
		No	Yes			No	Yes	
Non-drinking	19,094	18,698 (98.0)	396 (2.0)	<0.001	21,528	20,711 (96.1)	817 (3.9)	<0.001
Non-BDE	14,320	13,877 (97.0)	443 (3.0)		11,785	10,985 (93.0)	800 (7.0)	
BDE	3,241	3,015 (93.1)	226 (6.9)		2,467	2,128 (85.8)	339 (14.2)	

Values are presented as number (%).

**Table 3.** Binge drinking experience and suicide attempts in binge drinkers, by age

Age (yr)	Total (n)	Male		p-value	Total (n)	Female		p-value
		No	Yes			No	Yes	
12, 13	83	68 (82.9)	15 (17.1)	<0.001	114	83 (69.4)	31 (30.6)	<0.001
14	179	151 (84.6)	28 (15.4)		185	138 (73.4)	47 (26.6)	
15	452	418 (92.2)	34 (7.8)		342	275 (78.5)	67 (21.5)	
16	804	765 (95.4)	39 (4.6)		616	546 (89.6)	70 (10.4)	
17	1,120	1,069 (95.4)	51 (4.6)		822	748 (91.0)	74 (9.0)	
18	523	495 (94.3)	28 (5.7)		323	298 (92.1)	25 (7.9)	

Values are presented as number (%).

**Table 4.** Relationship between binge drinking experience (BDE) and suicide attempts

Variable	Male		Female	
	Crude OR (95% CI)	Adjusted OR (95% CI)^1^	Crude OR (95% CI)	Adjusted OR (95% CI)^1^
Non-drinking	1.00 (reference)	1.00 (reference)	1.00 (reference)	1.00 (reference)
Non-BDE	1.52 (1.32, 1.76)	1.04 (0.88, 1.23)	1.88 (1.70, 2.08)	1.21 (1.07, 1.37)
BDE	3.63 (3.09, 4.27)	1.63 (1.28, 2.09)	4.12 (3.59, 4.73)	1.79 (1.47, 2.19)

OR, odds ratio; CI, confidence interval.

1 Age, health behavior factors (smoking experience, substance experience), mental health factors (stress, depression experience, suicidal ideation).

**Table 5.** Relationship between binge drinking experience (BDE) and suicide attempts, by age

Age (yr)	BDE vs. non-BDE			
	Male		Female	
	Crude OR (95% CI)	Adjusted OR (95% CI)^1^	Crude OR (95% CI)	Adjusted OR (95% CI)^1^
12, 13	3.71 (2.06, 6.70)	3.97 (1.57, 10.03)	2.75 (1.74, 4.35)	1.49 (0.83, 2.69)
14	4.93 (3.09, 7.89)	3.21 (1.80, 5.71)	2.56 (1.76, 3.70)	1.16 (0.72, 1.87)
15	2.90 (1.87, 4.50)	1.50 (0.93, 2.44)	4.11 (3.00, 5.63)	2.66 (1.79, 3.96)
16	2.37 (1.64, 3.41)	1.46 (0.93, 2.28)	2.85 (2.06, 3.93)	1.61 (1.09, 2.36)
17	2.39 (1.71, 3.33)	1.53 (0.98, 2.38)	2.36 (1.78, 3.12)	1.36 (0.95, 1.95)
18	2.79 (1.49, 5.25)	2.32 (1.07, 5.03)	2.84 (1.66, 4.86)	1.17 (0.59, 2.31)

OR, odds ratio; CI, confidence interval.

1 Health behavior factors (smoking experience, substance experience), mental health factors (stress, depression experience, suicidal ideation).

## SUPPLEMENTARY MATERIALS

Supplementary Material: Korean version is available at (http://www.e-epih.org/).
